# Depth-specific characterization of bone microarchitecture in the femoral head: implications for osteoarthritis and osteoporosis research

**DOI:** 10.1186/s13075-026-03943-2

**Published:** 2026-11-03

**Authors:** Alexander Simon, Assil-Ramin Alimy, Johannes Krug, Björn Busse, Frank Timo Beil, Michael Amling, Felix N. von Brackel, Tim Rolvien

**Affiliations:** 1 Department of Trauma and Orthopaedic Surgery, Division of Orthopaedics, University Medical Center Hamburg-Eppendorf, Martinistrasse 52, 20246 Hamburg, Germany; 2 Department of Osteology and Biomechanics, University Medical Center Hamburg-Eppendorf, Martinistrasse 52, 20246 Hamburg, Germany

**Keywords:** Hip osteoarthritis, Osteoporosis, Subchondral bone, bone microarchitecture, HR-pQCT

## Abstract

**Background:**

Osteoarthritis (OA) and osteoporosis (OP) are highly prevalent musculoskeletal diseases characterized by cartilage loss and increased subchondral bone formation and by systemic bone mass reduction, respectively. The femoral head is commonly examined in both OA and OP research, yet both conditions may coexist in the same patient, making it unclear how local cartilage degeneration and systemic bone mineral density (BMD), as a marker of OP-related skeletal status, contribute to bone microarchitectural changes at different depths.

**Methods:**

Femoral heads were obtained from 30 patients undergoing primary total hip arthroplasty for hip OA. Ex vivo high-resolution peripheral quantitative computed tomography (HR-pQCT) was performed on the entire femoral head, followed by computational segmentation of the subchondral mineralized plate (SMP) and five concentric trabecular regions. Depth-specific bone microarchitectural parameters were analyzed in relation to histologically assessed cartilage degeneration and systemic BMD measured by dual-energy X-ray absorptiometry (DXA).

**Results:**

The cohort had a mean age of 75.4 ± 9.5 years, 53.3% were female, and patients spanned the spectrum of normal BMD (36.7%), osteopenia (46.7%), and osteoporosis (16.7%). Trabecular volumetric tissue mineral density (Tb.vTMD), bone volume to tissue volume (BV/TV), and trabecular thickness (Tb.Th) showed an initial decrease in the superficial trabecular regions, followed by a progressive increase toward deeper regions, whereas cyst-like void volume continuously decreased with depth and almost completely disappeared beyond 9.84 mm in depth. Cartilage degeneration independently predicted subchondral bone microstructure within the SMP and in superficial to intermediate trabecular regions (up to 13.12 mm in depth). Specifically, higher OARSI grades were associated with higher SMP.Th, Tb.vTMD, BV/TV, and Tb.Th. Systemic BMD T-score emerged as an independent predictor of trabecular bone parameters across all depths and represented the sole determinant of BV/TV and Tb.Th in the deepest trabecular region (beyond 13.12 mm depth), while cartilage degeneration was not associated with trabecular bone parameters in the deepest trabecular region.

**Conclusions:**

Our findings showcase spatially distinct contributions of cartilage degeneration and systemic skeletal status to subchondral bone remodeling, identifying depth as a critical determinant in the analysis of osteochondral alterations in hip OA. Specifically, our findings emphasize that systemic BMD should be considered a covariate in OA research and that deep regions of the femoral head should be used for OP research to minimize potential confounding by local OA-related effects.

**Supplementary Information:**

The online version contains supplementary material available at https://doi.org/10.1186/s13075-026-03943-2.

## Introduction

Osteoarthritis (OA) and osteoporosis (OP) are among the most prevalent musculoskeletal diseases worldwide. OA leads to progressive loss of joint function [1], whereas OP is characterized by increased bone fragility [2]. Together, these conditions affect around one billion people globally [3, 4]. Although OA and OP are generally regarded as mechanistically distinct disorders of cartilage and bone, respectively, OA is accompanied by marked alterations in subchondral bone, characterized by local bone loss in early disease stages and osteosclerosis in advanced stages [5]. Importantly, OA and OP are not mutually exclusive and may coexist in the same patient [6–8], complicating the distinction between local OA-related remodeling and systemic OP-related bone loss. The complexity of this relationship is reflected in apparently divergent observations: higher systemic BMD has been associated with a greater prevalence of radiographic OA and has therefore been proposed as a potential risk factor for OA [9, 10], whereas concomitant OP may adversely affect subchondral bone and aggravate cartilage degeneration in patients with established OA [6].

The proximal femur is a major site of OA-related joint degeneration [11] and OP-related fragility fractures [12]. Consequently, the femoral head, routinely resected during hip arthroplasty to treat end-stage OA or femoral neck fractures, is widely analyzed to study both diseases. Numerous imaging and histological studies have investigated femoral head bone changes in hip OA [13–17] and OP [18, 19], and several studies have considered both conditions jointly [6, 20, 21]. However, while cartilage degeneration in OA is accompanied by substantial subchondral bone formation and increased local bone mass [5], it remains unclear to what depth these OA-related alterations extend into the femoral head. Determining this spatial extent is critical for study design: OP-focused analyses should ideally minimize OA-related influences, whereas investigations of OA, particularly of subchondral bone, should be conducted in regions least affected by systemic changes in bone mass. In this context, we have previously shown that systemic bone mineral density (BMD) exerts a negligible influence on the subchondral mineralized plate (SMP) [5, 22]. Li et al. reported regional and compartmental differences in subchondral bone microarchitecture and mineralization between osteoarthritic and osteoporotic femoral heads [23]. However, none of these studies quantitatively determined how far alterations associated with local cartilage degeneration extend into the trabecular bone or how the relative contributions of local cartilage degeneration and systemic BMD vary with depth.

In the present study, we therefore analyzed human femoral heads using high-resolution peripheral quantitative computed tomography (HR-pQCT) with a depth-specific approach. Microarchitectural parameters were subsequently interpreted in relation to local OA severity, assessed histologically, and systemic bone mass, determined by dual-energy X-ray absorptiometry (DXA). This integrative framework aims to provide guidance on the anatomical depth at which bone analyses should be performed when investigating OA- or OP-related changes.

## Methods

### Study cohort and specimen collection

Thirty femoral heads were obtained from 30 patients undergoing primary total hip arthroplasty (THA) for advanced hip OA. Hospital reports and preoperative imaging were screened to exclude individuals with hip pathologies other than primary OA (e.g., osteonecrosis, stress fracture, hip dysplasia) and conditions that could potentially compromise overall skeletal integrity (e.g., active cancer, diabetes, genetic bone diseases, glucocorticoid treatment, or periods of immobilization). Sex, age, and body mass index (BMI) were recorded systematically for all patients based on preoperative clinical documentation. Femoral heads were collected intraoperatively immediately after resection and fixed in 3.7% formaldehyde as reported previously [5]. The study was approved by the local ethics committee (Ethikkommission Arztekammer Hamburg, WF-020/20) and conducted in accordance with the Declaration of Helsinki.

### Clinical imaging and assessment of systemic bone mineral density

Standardized routine standing hip radiographs were obtained preoperatively and used to assess radiographic OA severity according to Kellgren-Lawrence grading [24]. In addition, the femoral neck-shaft angle was determined and classified as coxa vara (≤ 124°), normal alignment (125°-135°), or coxa valga (≥ 136°). Systemic BMD was evaluated preoperatively using DXA (Lunar Prodigy, GE Healthcare, Madison, WI, USA). Measurements were performed at the lu​mbar spine (L1–L4) and both hips whenever feasible. DXA scans were carefully reviewed, and the measurement regions were positioned to avoid the inclusion of marginal osteophytes as far as technically possible. Calibration scans were conducted daily in accordance with the manufacturer’s recommendations for DXA quality assurance and the guidance of the International Society for Clinical Densitometry [25]. Bone mineral density was expressed as T-scores, representing the number of standard deviations from the mean BMD of a sex-matched reference population of healthy adults aged 20–40 years. Stratification into normal BMD (T-score ≥ −1.0), osteopenia (−1.0 > T-score >​ −2.5), or osteoporosis (T-score ≤ −2.5) was performed according to World Health Organization (WHO) guidelines [26].

### Ex vivo high-resolution peripheral quantitative computed tomography (HR-pQCT) and computationa​l segmentation

To assess three-dimensional subchondral and trabecular bone microarchitecture as well as volumetric tissue mineral density (vTMD), the entire femoral head was scanned ex vivo using HR-pQCT (XtremeCT, Scanco Medical AG, Brüttisellen, Switzerland). All specimens were scanned using a standardized protocol (60 kVp, 900 µA, 100 ms integration time, isotropic voxel size of 41 µm). Image analysis was performed using a custom computational workflow implemented in XamFlow (Lucid AG, Zurich, Switzerland). Briefly, the subchondral mineralized plate (SMP) and trabecular compartment were isolated following established workflows for long bones applied to the femoral head re-oriented to the femoral neck axis to align the femoral neck axis with the axial cutting planes [27]. Subsequently, the approach involves a grey value threshold (257.2 mg HA/cm^3^) to separate mineralized tissue from background/soft tissue. The resulting representation of mineralized tissue was subsequently closed, and remaining cavities were filled, resulting in a mask of the femoral head, defining the outer boundary of the SMP. Additionally, a second threshold (157.2 mg HA/cm^3^) was applied to the original image. The resulting binarization was combined with the previously generated mask to achieve a representation of the bone marrow. By morphological operations applied to the binary bone marrow volume, trabeculae were removed, resulting in a maented into five conc

sk of the trabecular compartment that was smoothed at its boundaries using a Gaussian filter for blur, followed by another threshold for smoothing. Finally, the trabecular compartment mask was subtracted from the previous femoral head mask, resulting in a representation of the SMP.

The SMP (R0) was defined as the mineralized layer directly beneath the articular cartilage and comprised the calcified cartilage and the underlying subchondral bone plate, which cannot be reliably distinguished at 41 µm HR-pQCT isometric voxel size [28]. Starting from the inner boundary of R0, the trabecular bone was segmented into five concentric regions (R1 to R5) of equal thickness (80 voxels × 41 µm slice thickness = 3.28 mm region thickness) towards the center of the femoral head, to enable a systematic assessment of depth-specific alterations in bone microarchitecture. Accordingly, all reported depth intervals refer exclusively to trabecular depth and do not include the variable thickness of R0. The regions extended from superficial to deeper trabecular bone, specifically from 0 to 3.28 mm (R1), 3.28 to 6.56 mm (R2), 6.56 to 9.84 mm (R3), 9.84 to 13.12 mm (R4), and 13.12 to 16.40 mm (R5) beneath the inner boundary of R0. Although an additional central region was present in larger femoral heads, the analysis was restricted to R1 to R5 because all specimens exhibited at least a diameter of 32.80 mm, thereby ensuring consistent comparisons across depths throughout the cohort.

For the quantification of cyst-like voids, trabecular separation was thresholded at 1.94 mm to distinguish normal intertrabecular spaces from larger focal voids. We have thereby chosen a more conservative threshold than others [29, 30]. The threshold was selected by visually comparing the resulting segmentations to avoid selecting large intertrabecular, structural volumes and was subsequently applied uniformly to all specimens and trabecular regions. Regions exceeding this threshold were classified as cyst-like voids, and their volume was reported as cyst-like void volume (CLV.V). Because HR-pQCT depicts the absence of mineralized tissue but cannot determine the contents or histological identity of these voids, they were not considered confirmed subchondral cysts.

HR-pQCT-derived parameters included SMP thickness (SMP.Th). For each concentric trabecular region, region volume, trabecular volumetric tissue mineral density (Tb.vTMD), bone volume to tissue volume (BV/TV), trabecular number (Tb.N), trabecular thickness (Tb.Th), trabecular separation (Tb.Sp), and cyst-like void volume (CLV.V) were quantified according to current guidelines [31]. Tb.Th and Tb.Sp were determined directly from the segmented three-dimensional trabecular structure by a sphere fitting approach [32].

### Histological assessment of cartilage degeneration

Undecalcified femoral head specimens were used for histological evaluation of cartilage degeneration, as described previously [5]. Following standardized coronal sectioning of the proximal half of each femoral head through the fovea capitis using a diamond-coated saw, the sections were divided into three regions of equal size. Specimens were dehydrated, embedded undecalcified in methyl methacrylate, and sectioned at a thickness of 4 µm. Sections were stained with Safranin O-Light Green (Supplementary Table 1) to assess cartilage morphology and integrity. Mean cartilage thickness (Cg.Th) was quantified using OsteoMeasure (OsteoMetrics, Atlanta, GA, USA) in accordance with the guidelines of the American Society for Bone and Mineral Research [33]. Histopathological cartilage degeneration was determined using the OARSI grading system [34]. Each region was evaluated separately, and the mean values of the three regions were used for subsequent analyses. The histological regions were not directly spatially registered to the three-dimensional concentric HR-pQCT regions, which encompassed the entire femoral head.

### Statistical analysis

Statistical analysis was performed using SPSS Statistics 30.0.0 (IBM, Armonk, NY, USA) and GraphPad Prism 10.6.1 (GraphPad Software, San Diego, CA, USA). Continuous variables are reported as mean ± standard deviation, whereas categorical variables are presented as absolute numbers and percentages. Normality of data distribution was assessed using the Shapiro–Wilk test. Depth-specific differences between adjacent regions were analyzed using repeated measures one-way analysis of variance (ANOVA) with Holm-Šidák test for normally distributed data and Friedman test with Dunn’s test for non-normally distributed data. Corresponding effect sizes are reported as Cohen’s d (0.2 ≙ small, 0.5 ≙ medium, 0.8 ≙ large effect size). Associations of depth-specific bone parameters with cartilage degeneration (OARSI grade, Cg.Th) and systemic BMD (affected hip T-score, lowest T-score) were assessed using Pearson correlation for normally distributed variables and Spearman rank correlation for non-normally distributed variables. Correlation coefficients r and corresponding *p*-values are reported. The correlation analyses were considered exploratory, and no adjustment for multiple comparisons was applied. Multiple linear regression analyses were performed to identify independent predictors of depth-specific bone microarchitecture. In addition to demographic variables (sex (0 = female, 1 = male), age, and BMI), cartilage degeneration (OARSI grade) and systemic BMD (affected hip T-score) were included as independent variables using backward elimination with a removal threshold of *p* ≥ 0.1. The affected hip T-score was selected because it provided the site-matched clinical DXA measure corresponding most closely to the operated proximal femur from which the examined femoral head was obtained and was therefore considered most clinically relevant in the context of local bone strength and potential perioperative and periprosthetic fracture susceptibility. Model assumptions were tested and confirmed. Adjusted R^2^ values, standardized regression coefficients β, and corresponding *p*-values are reported for all models. Statistical significance was defined as *p* < 0.05.

## Results

### Characteristics of the study cohort

The study cohort comprised 30 patients who underwent primary THA, with a mean age of 75.4 ± 9.5 years (range 55 to 88 years) (Table 1). The proportions of female and male patients were comparable (53.3% female), and the mean BMI was 27.3 ± 2.9 kg/m^2^ (range 21.9 to 33.9 kg/m^2^). Preoperative radiographic assessment demonstrated advanced osteoarthritic changes, with a mean Kellgren-Lawrence grade of 3.1 ± 0.8 (range 2 to 4), which was reflected by a mean cartilage thickness of 0.7 ± 0.4 mm (range 0.1 to 1.6 mm) and a mean OARSI grade of 4.0 ± 1.1 (range 2 to 6) on histological examination. With respect to systemic BMD, the study cohort was distributed across normal BMD (36.7%), osteopenia (46.7%), and osteoporosis (16.7%), with a mean lowest T-score of −1.1 ± 1.4 (range −3.7 to 1.4). In addition, assessment of proximal femoral geometry revealed a mean femoral neck-shaft angle of 128.6 ± 8.2°, with 33.3% classified as coxa vara, 40.0% as normal alignment, and 26.7% as coxa valga.

**Table 1:** Characteristics of the study cohort

	**Patients** (*n* = 30)			
Parameter	Mean	SD	Min	Max
**Demographics**				
Female (%)	53.3			
Age (years)	75.4	9.5	55	88
BMI (kg/m^2^)	27.3	2.9	21.9	33.9
**DXA**				
Affected Hip T-score	−0.9	1.3	−3.7	1.9
Lowest T-score	−1.1	1.4	−3.7	1.4
Normal BMD (*T* ≥ −1.0)	​11 of 30 (36.7%)			
Osteopenia (−1.0 > *T* > −2.5)	14 of 30 (46.7%)			
Osteoporosis (*T* ≤ −2​.5)	5 of 30 (16.7%)			
**Kellgren-Lawrence**				
Grade	3.1	0.8	2	4
Grade 0	0 of 30 (0.0%)			
Grade 1	0 of 30 (0.0%)			
Grade 2	9 of 30 (30.0%)			
Grade 3	10 of 30 (33.3%)			
Grade 4	11 of 30 (36.7%)			
**Cartilage Thickness**				
Cg.Th (mm)	0.7	0.4	0.1	1.6
**OARSI**				
Grade	4.0	1.1	2	6
0–2	3 of 30 (10.0%)			
3–4	19 of 30 (63.3%)			
5–6	8 of 30 (26.7%)			
**Neck-Shaft Angle**				
Neck-Shaft Angle	128.6	8.2	114	146
Coxa vara (≤ 124°)	1​0 of 30 (33.3%)			
Normal (125°-135°)	12 of 30 (40.0%)			
Coxa valga (≥ 136°)	8 of 30 (26.7%)			

Data are presented as mean ± standard deviation with corresponding minimum and maximum values. For BMD categories (based on lowest T-score of any site), Kellgren-Lawrence grades, OARSI grades and femoral neck-shaft angle categories absolute values with corresponding percentages are presented

Abbreviations: BMD, Bone mineral density. BMI, Body mass index. Cg.Th, Cartilage thickness. DXA, Dual-energy X-ray absorptiometry. Max, Maximum. Min, Minimum. SD, Standard deviation

### Depth-specific alterations of subchondral bone microarchitecture

**Fig. 1:**
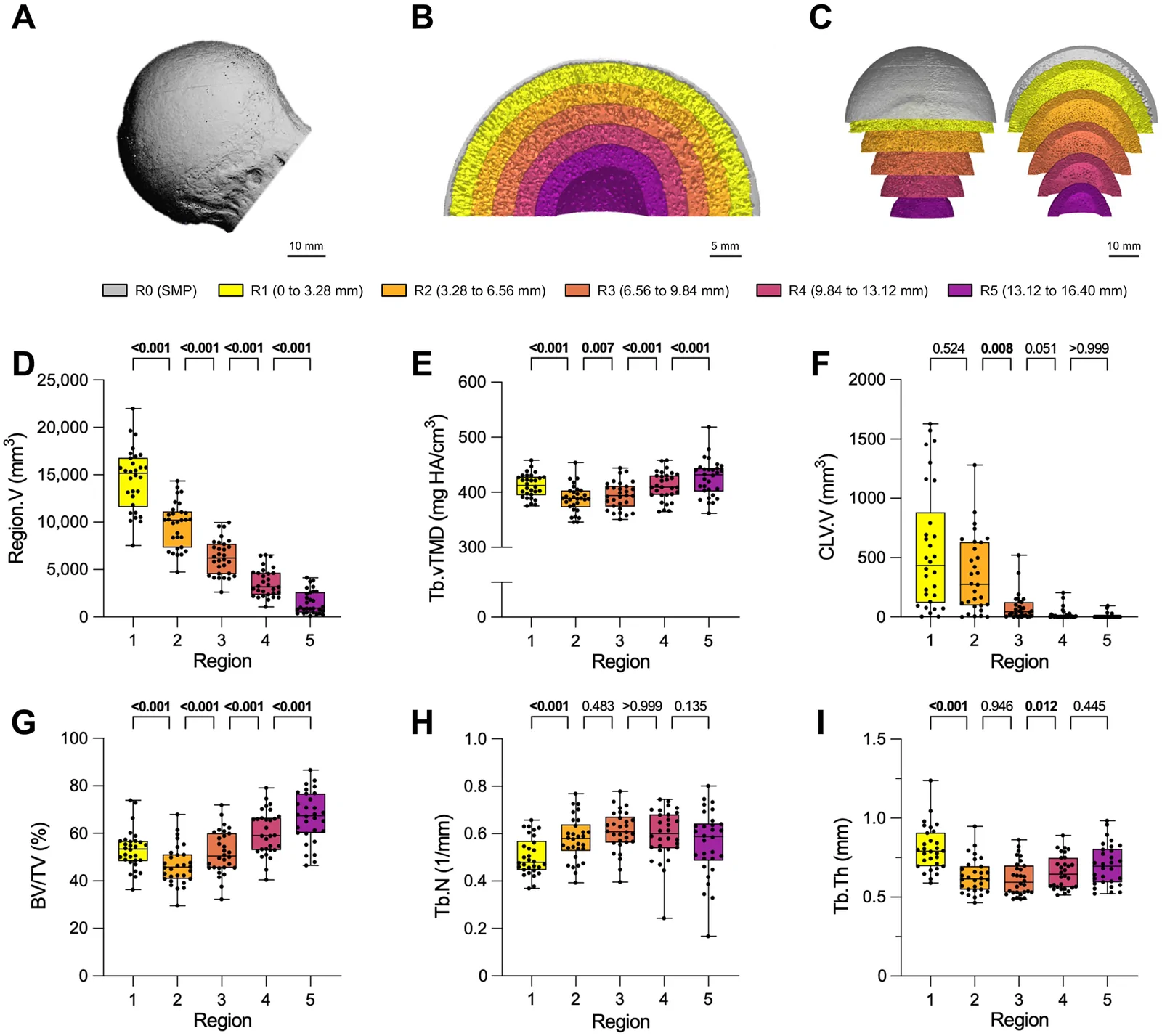
Depth-specific alterations of subchondral bone microarchitecture. (**A**) Three-dimensional reconstruction of a representative femoral head derived from ex vivo HR-pQCT imaging. (**B**) cross-sectional view illustrating the computational segmentation approach of the subchondral bone with concentric trabecular layers (i.e., regions), each with a uniform thickness of 3.28 mm, defined from the inner boundary of the subchondral mineralized plate (SMP) towards the central trabecular bone. (**C**) Three-dimensional visualization of the segmented subchondral bone with concentric trabecular layers. (**D-I**) Comparison of HR-pQCT-derived parameters across the five depth-specific trabecular regions, including (**D**) region volume (Region.V), (**E**) trabecular volumetric tissue mineral density (Tb.vTMD), (**F**) cyst-like void volume (CLV.V), (**G**) bone volume to tissue volume (BV/TV), (**H**) trabecular number (Tb.N), and (**I**) trabecular thickness (Tb.Th)

Following three-dimensional ex viv​o HR-pQCT imaging and segmentation of the femoral head into the SMP and concentric trabecular regions (Fig. 1A–C), the SMP exhibited a mean thickness of 1.694 ± 0.411 mm. Below the SMP, a progressive decrease in Region.V was observed with increasing depth (all *p* < 0.001, Fig. 1D). Tb.vTMD showed depth-specific differences, with an initial decrease from the superficial trabecular region to the adjacent region (R1 vs. R2, *p* < 0.001), followed by a gradual increase toward deeper regions, reaching the highest values in the deepest evaluated region (all *p* < 0.01, Fig. 1E). CLV.V was comparable in the two most superficial trabecular regions (R1 vs. R2, *p* = 0.524). Nevertheless, it decreased continuously toward deeper regions, with a marked reduction observed between the intermediate regions (R2 vs. R3, *p* = 0.008, d = 0.98) (Fig. 1F). In a pattern comparable to Tb.vTMD, BV/TV initially declined in the superficial regions (R1 vs. R2, *p* < 0.001) and subsequently increased steadily toward deeper trabecular regions (all *p* < 0.001) (Fig. 1G). With respect to trabecular microarchitecture, Tb.N increased from R1 to R2 (*p* < 0.001) and remained largely constant across the deeper regions (R2 to R5, *p* > 0.1) (Fig. 1H). In contrast, Tb.Th decreased from R1 to R2 (*p* < 0.001) and subsequently increased toward deeper regions with statistically significant differences observed between R3 and R4 (*p* = 0.012) (Fig. 1I). Additional data on bone microarchitecture parameters and group comparisons are provided in Supplementary Table 2.

### Depth-specific associations of cartilage degeneration and systemic bone mineral density with subchondral bone microarchitecture

**Fig. 2:**
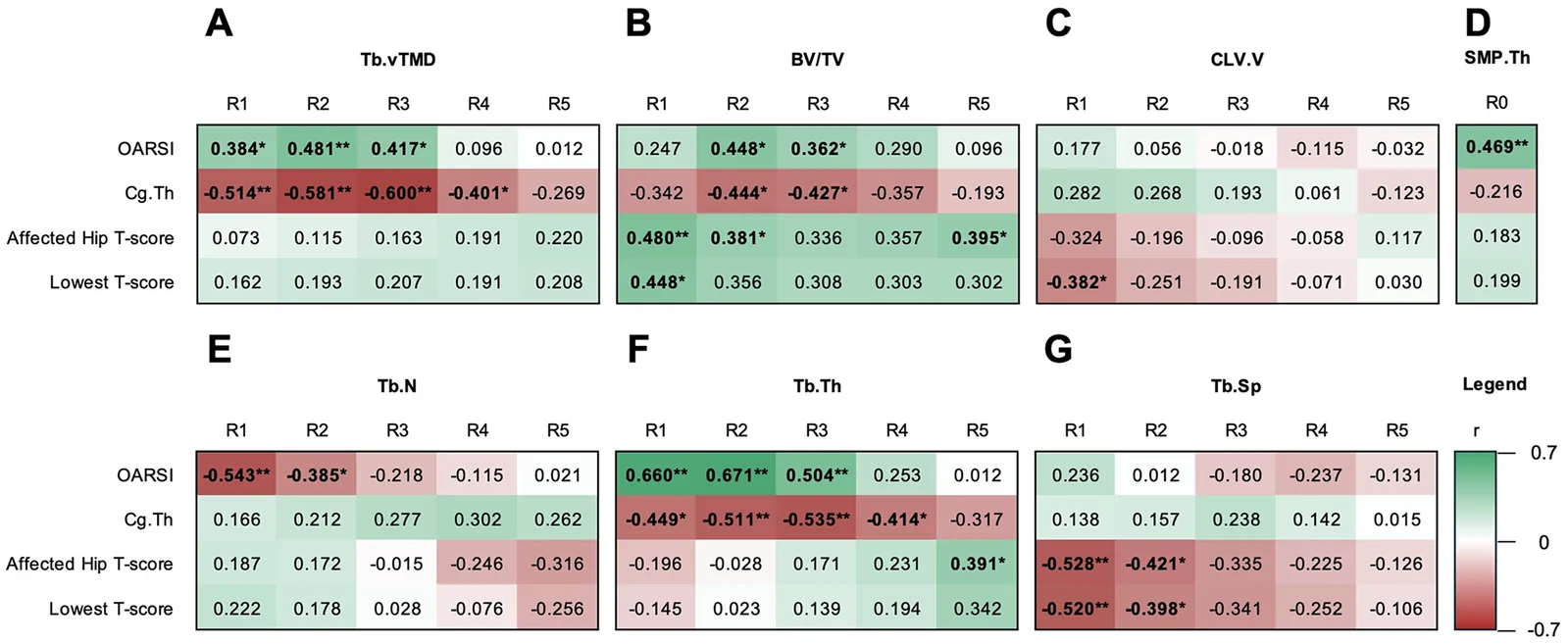
Depth-specific associations of cartilage degeneration and systemic bone mineral density with subchondral bone microarchitecture. (**A-G**) Heatmaps illustrating correlations of (**A**) trabecular volumetric tissue mineral density (Tb.vTMD), (**B**) bone volume to tissue volume (BV/TV), (**C**) cyst-like void volume (CLV.V), (**D**) subchondral mineralized plate thickness (SMP.Th), (**E**) trabecular number (Tb.N), (**F**) trabecular thickness (Tb.Th), and (**G**) trabecular separation (Tb.Sp) with local cartilage degeneration (OARSI grade and cartilage thickness) and DXA-derived systemic bone mineral density (affected hip T-score and lowest T-score of any site) across the five concentric trabecular regions (R1 to R5) and the subchondral mineralized plate (R0). Color coding represents correlation coefficients r, with green indicating positive and red indicating negative correlations. Exact *r*-values are displayed and numbers in bold indicate statistical significance (* *p* < 0.05, ** *p* < 0.01)

Tb.vTMD showed significant associations with cartilage degeneration in the superficial and intermediate regions (R1 to R4), with positive correlations with OARSI grade in R1 to R3 (all *r* > 0.3, *p* < 0.05), and negative correlations with cartilage thickness in R1 to R4 (all *r* < −0.4, *p* < 0.05) (Fig. 2A). In contrast, no statistically significant associations were observed between Tb.vTMD and cartilage degeneration in the deepest investigated region (R5, *p* > 0.05), nor were any significant associations detected between Tb.vTMD and systemic BMD across all regions (R1 to R5, *p* > 0.05). BV/TV exhibited a distinct depth-specific association pattern (Fig. 2B). Significant correlations of BV/TV with cartilage degeneration were observed in the superficial to intermediate trabecular regions (R2 to R3, *p* < 0.05), whereas no statistically significant associations were detected in the most superficial trabecular region or in the deeper trabecular regions (R1 and R4 to R5, *p* > 0.05). Conversely, BV/TV was significantly associated with systemic BMD, with positive correlations observed in the most superficial trabecular regions (R1 to R2, *p* < 0.05) as well as in the deepest trabecular region (R5, *p* < 0.05). With respect to CLV.V, only a negative correlation with systemic BMD was observed in the most superficial region (R1, *p* < 0.05), whereas no significant associations were detected in deeper regions or with cartilage degeneration (Fig. 2C). SMP.Th was positively associated with cartilage degeneration (OARSI grade, *r* > 0.4, *p* < 0.05) (Fig. 2D), while Tb.N was negatively associated with cartilage degeneration in the superficial regions (R1 to R2, *p* < 0.05) (Fig. 2E). Similar to Tb.vTMD, Tb.Th showed pronounced associations with cartilage degeneration across the superficial and intermediate trabecular regions (R1 to R4, *r* > 0.4, *p* < 0.05), with increasing Tb.Th observed with advancing cartilage degeneration (Fig. 2F). Systemic BMD correlated with Tb.Th only in the deepest investigated region (R5, *p* < 0.05). Tb.Sp did not correlate with cartilage degeneration but was significantly associated with systemic BMD in the superficial trabecular regions (R1 to R2, *p* < 0.05) (Fig. 2G).

### Depth-specific independent predictors of subchondral bone microarchitecture

**Fig. 3:**
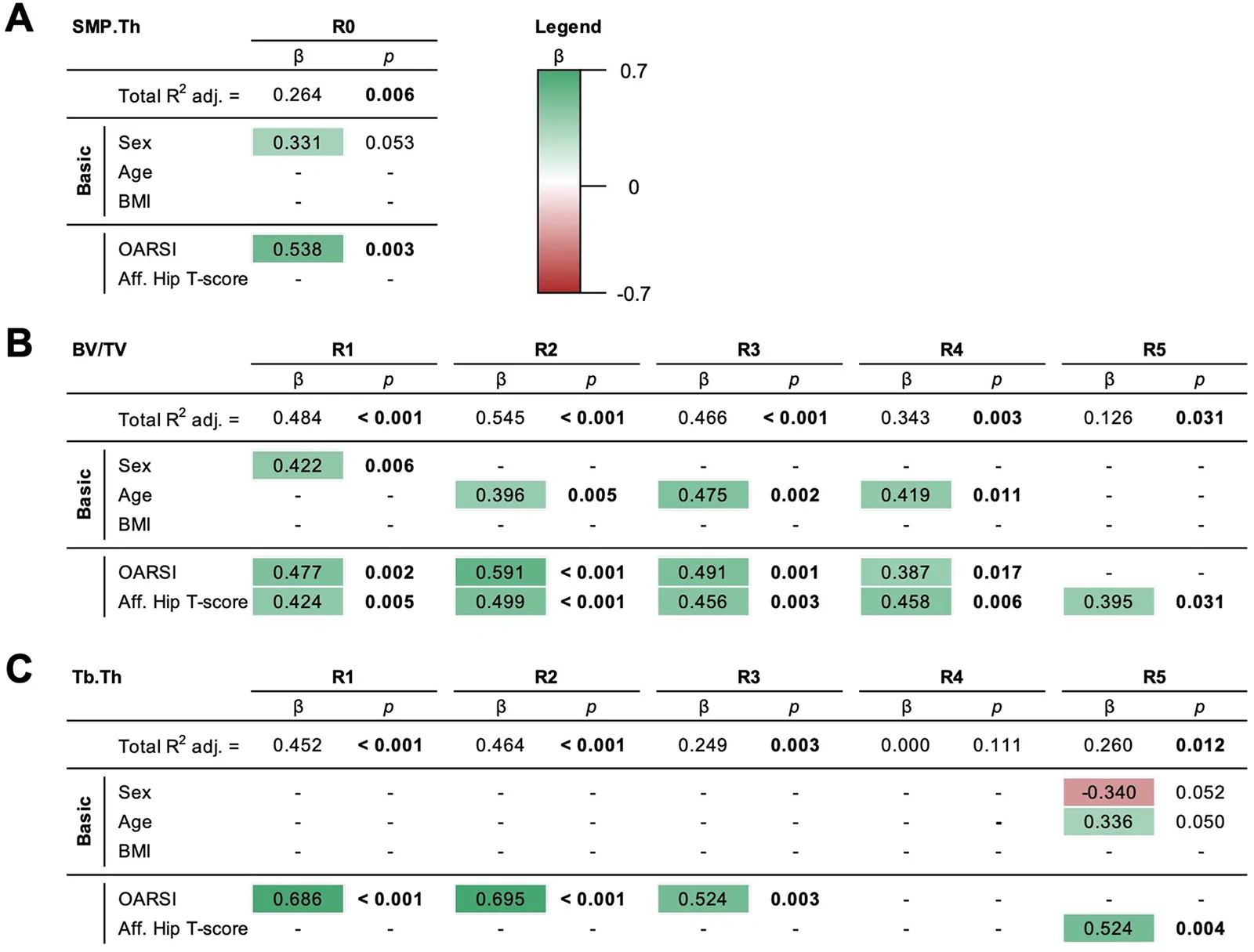
Depth-specific independent predictors of subchondral bone microarchitecture. (**A-C**) Heatmaps derived from multiple linear regression models (backward elimination) to identify independent predictors of (**A**) subchondral mineralized plate thickness (SMP​.Th), and (**B**) bone volume to tissue volume (BV/TV), as well as (**C**) trabecular thickness (Tb.Th) across the five concentric trabecular regions (R1 to R5). For each model, adjusted coefficients of determination R^2^ with corresponding *p*-values and standardized regression coefficients β with corresponding *p*-values are shown. Sex (0 = female, 1 = male), age, body mass index (BMI), OARSI grade and affected hip T-score were included as variables in all models. Color coding indicates standardized regression coefficients β, with green indicating positive and red indicating negative associations. Numbers in bold indicate statistical significance (*p* < 0.05)

Multiple linear regression analyses were performed to identify independent predictors of depth-specific bone microarchitecture while accounting for demographic variables, cartilage degeneration, and systemic BMD. Sex, age, BMI, OARSI grade, and affected hip T-score were included as candidate predictors. At the level of SMP, cartilage degeneration proved to be the only independent predictor of SMP.Th, with higher OARSI grades associated with increased SMP.Th (β = 0.538, *p* = 0.003) (Fig. 3A). For BV/TV, cartilage degeneration was an independent predictor in the superficial and intermediate trabecular regions (R1 to R4, all β > 0.38, *p* < 0.02) (Fig. 3B). In addition, systemic BMD, as reflected by affected hip T-score, was an independent predictor across all trabecular regions (R1 to R5, all β > 0.39, *p* < 0.05). Sex (R1) and age (R2 to R4) also contributed as independent predictors. With regard to Tb.Th, cartilage degeneration emerged as the predominant independent predictor in the superficial and intermediate trabecular regions (R1 to R3), with higher OARSI grades associated with increased Tb.Th (all β > 0.52, *p* < 0.01) (Fig. 3C). In contrast, systemic BMD was identified as the sole independent predictor in the deepest trabecular region (R5, β = 0.524, *p* = 0.004). Additional multiple linear regression models for Tb.vTMD, Tb.N, Tb.Sp, and CLV.V are provided in Supplementary Figure 1. In supplementary analyses using the lowest T-score across available DXA sites instead of the affected hip T-score, the resulting models were largely comparable, with similar results in the superficial and intermediate regions (Supplementary Fig. 2). In R5, the lowest T-score remained the only statistically significant predictor of Tb.Th (β = 0.452, *p* = 0.015), whereas the association with BV/TV was not retained.

## Discussion

Bone mass is influenced by systemic and local factors, with OP being the most common generalized bone loss syndrome and OA representing a common stimulus for local subchondral bone formation (i.e., osteosclerosis). In the femoral head, both phenomena overlap, so that precise knowledge of region-specific (i.e., depth-specific) influences is essential to identify target regions for studying either OP or OA. Here, using HR-pQCT imaging of human femoral heads with varying OA grades, we demonstrated how various bone microarchitectural parameters change in depth-specific layers from superficial to deeper trabecular regions and, most importantly, how these parameters are influenced by systemic bone mass, assessed by gold standard DXA, and local OA grade, assessed by histological cartilage measurements. We found that local OA grade strongly influenced subchondral bone volume fraction (BV/TV), particularly via trabecular thickening, extending to R4 (13.12 mm in depth). While cartilage degeneration emerged as an independent predictor of subchondral bone microarchitecture in superficial and intermediate regions, but not in the deepest region (beyond 13.12 mm in depth), systemic BMD was associated with subchondral bone micro architecture a​cross all depths and represented the sole independent predictor of BV/TV and Tb.Th in the deepest trabecular region. These results highlight an influence of local OA stimuli on subchondral microstructure in deeper regions than previously reported [5], as well as an influence of BMD alone in the deepest region, suggesting that OP research on femoral heads with any degree of OA should focus on the deep trabecular regions beyond 13.12 mm to prevent the influence of OA on the bone microarchitecture.

Although femoral heads obtained from patients undergoing THA for OA are frequently used in human bone research, OA is known to induce pronounced, spatially heterogeneous alterations of bone microarchitecture, particularly in the subchondral region [5, 14, 35]. These local changes, including sclerosis and altered trabecular organization, can confound analyses aimed at assessing systemic bone status or osteoporosis-related phenotypes. Here, we determined both cartilage thickness and OARSI grade histologically and correlated them with depth-specific bone microarchitecture assessed by HR-pQCT. We found strong independent effects of cartilage degeneration on SMP.Th (R0) and on several trabecular bone parameters across superficial and intermediate regions, including Tb.vTMD and Tb.Th (R1 to R3; up to 9.84 mm in depth), BV/TV (R1 to R4; up to 13.12 mm in depth), Tb.N (R1 to R2; up to 6.56 mm in depth), and Tb.Sp (R3; 6.56 to 9.84 mm in depth). Spatial OA-specific effects were also evident in the formation of subchondral cyst-like voids, which decreased significantly from region 2 to region 3 and had almost completely disappeared in region 4 (beyond 9.84 mm in depth).

While OA induces local bone microarchitectural changes, we additionally found a significant effect of systemic BMD on femoral head microarchitecture in all five regions. Therefore, it can be concluded that osteoarthritic femoral heads for OP research should ideally include and analyze the deep region and that trabecular microarchitecture in the superficial regions is susceptible to confounding for OP-specific questions due to the strong local influence of OA. Our approach of identifying an optimal depth minimally influenced by OA aligns with previous evidence that deeper trabecular regions may be less perturbed than subchondral trabecular bone [13]. A previous µ-CT study concluded that OA has pronounced effects on the bone microstructure of the femoral head in a regionalized and stage-specific manner with respect to cartilage degeneration [23]. However, an exact determination of how deep these effects reach was unavailable until now.

Despite the novelty of the present study, several limitations should be considered. First, the cross-sectional design does not allow causal inference regarding the sequence of cartilage degeneration and depth-specific subchondral bone alterations. Due to the nature of femoral head retrieval during THA, longitudinal assessment of identical anatomical regions over time is inherently not feasible. Consequently, disease progression can only be inferred indirectly based on spatial and histological gradients. Second, all femoral heads were obtained from patients undergoing THA for advanced hip OA. Although the cohort was evenly distributed across radiographic Kellgren–Lawrence grades 2 to 4, it does not represent early or preclinical OA. Consequently, the findings cannot be extrapolated to early disease. Third, although cartilage degeneration and thickness were assessed across three regions of a standardized coronal section, these two-dimensional histological regions were not directly spatially registered to the three-dimensional whole-head HR-pQCT regions. Accordingly, the observed relationships represent between-specimen associations rather than direct spatial co-localization. In addition, although high-resolution imaging was used, the combination of an isotropic voxel size of 41 µm and the applied segmentation approach limits the reliability of subchondral bone segmentation and cyst-like void detection. Consequently, the results may differ from histological evaluation and depend on the selected segmentation thresholds. Fourth, the limited cohort size and the inclusion of five candidate predictors may have affected the stability of the multivariable regression models and increased the risk of overfitting. However, the candidate variables were restricted to established demographic factors and the two primary parameters of interest. Furthermore, the exploratory correlation analyses were not corrected for multiple comparisons, increasing the risk of false-positive findings. Finally, while the present study provides detailed structural insights into depth-specific subchondral bone remodeling, it does not address the underlying molecular mechanisms. Future studies combining high-resolution imaging with molecular and cellular analyses are warranted to elucidate the cellular and biological pathways that mediate the spatially distinct effects of cartilage degeneration and systemic bone status.

In summary, our findings reveal a pronounced depth-specific dissociation between local OA-related remodeling and systemic skeletal influences within the femoral head. Cartilage degeneration primarily affects subchondral bone microarchitecture in superficial and intermediate regions, whereas systemic bone mineral density emerges as its sole determinant in the deepest trabecular layer, independent of local cartilage damage. These results underscore depth as a critical parameter in osteochondral analyses and provide practical guidance for the anatomical selection of target regions when investigating OA- or OP-related bone alterations in femoral heads.

## Electronic supplementary material

Below is the link to the electronic supplementary material.

Supplementary material 1

## Data Availability

The datasets used and analyzed during the present study are available from the corresponding author upon reasonable request.
