# Supplementary material for: Depth-specific characterization of bone microarchitecture in the femoral head: implications for osteoarthritis and osteoporosis research
Source: Arthritis Res Ther. 2026 Nov 3;28:194. doi: 10.1186/s13075-026-03943-2 (PMC13737963; doi:10.1186/s13075-026-03943-2)
Supplement: Supplementary file 1 [file 13075_2026_3943_MOESM1_ESM.docx]

**Supplementary material**

**Depth-specific characterization of bone microarchitecture in the femoral head: implications for osteoarthritis and osteoporosis research**

Alexander Simon, Assil-Ramin Alimy, Johannes Krug, Björn Busse, Frank Timo Beil, Michael Amling, Felix N. von Brackel and Tim Rolvien

**Supplementary Figure 1 Depth-specific independent predictors of additional subchondral bone microarchitecture parameters.** (A-D) Heatmaps derived from multiple linear regression models (backward elimination) to identify independent predictors of (A) trabecular volumetric tissue mineral density (Tb.vTMD), (B) trabecular number (Tb.N), (C) trabecular separation (Tb.Sp), and (D) cyst-like void volume (CLV.V) across the five concentric trabecular regions (R1-R5). For each model, adjusted coefficients of determination R^2^ with corresponding p-values and standardized regression coefficients β with corresponding p-values are shown. Sex (0 = female, 1 = male), age, body mass index (BMI), OARSI grade and affected hip T-score were included as variables in all models. Color coding indicates standardized regression coefficients β, with green indicating positive and red indicating negative associations. Numbers in bold indicate statistical significance (p < 0.05).

**Supplementary Figure 2 Depth-specific independent predictors of subchondral bone microarchitecture parameters.** (A-C) Heatmaps derived from multiple linear regression models (backward elimination) to identify independent predictors of (A) subchondral mineralized plate thickness (SMP.Th), and (B) bone volume to tissue volume (BV/TV), as well as (C) trabecular thickness (Tb.Th) across the five concentric trabecular regions (R1 to R5). For each model, adjusted coefficients of determination R2 with corresponding p-values and standardized regression coefficients β with corresponding p-values are shown. Sex (0 = female, 1 = male), age, body mass index (BMI), OARSI grade and lowest T-score were included as variables in all models. Color coding indicates standardized regression coefficients β, with green indicating positive and red indicating negative associations. Numbers in bold indicate statistical significance (p < 0.05).

**Supplementary Table 1 Protocol for Safranin O-Light Green Staining.**

| **Staining Procedure:** | |
| --- | --- |
| 1. | 2x5 min Xylene |
| 2. | 4x2 min Decreasing alcohol solutions (96%, 80%, 70%, 60%) |
| 3. | 2 min Aqua dest. |
| 4. | 10 min Weigert’s iron hematoxylin |
| 5. | 10 s Aqua dest. |
| 6. | 15 min Water |
| 7. | 10 s Aqua dest. |
| 8. | 3 min Light Green SF 0.1% |
| 9. | 10 s Acetic acid 1% |
| 10. | 6 min Safranin O solution 0.1% |
| 11. | 10 s Acetic acid 1% |
| 12. | 3x2 min Alcohol solutions (96%, 96%, 100%) |
| 13. | 2x5 min Xylene |
| 14. | Mount with a xylene-based mounting medium |

**Supplementary Table 2 Depth-specific changes in subchondral mineralized plate and trabecular bone microarchitecture.**

|  |  | **R0 / R1** (n = 30) | |  | **R2** (n = 30) | |  | **R3** (n = 30) | |  | **R4** (n = 30) | |  | **R5** (n = 30) | |  | **R1 vs. R2** | |  | **R2 vs. R3** | |  | **R3 vs. R4** | |  | **R4 vs. R5** | |
| --- | --- | --- | --- | --- | --- | --- | --- | --- | --- | --- | --- | --- | --- | --- | --- | --- | --- | --- | --- | --- | --- | --- | --- | --- | --- | --- | --- |
| HR-pQCT Parameter | | Mean | SD |  | Mean | SD |  | Mean | SD |  | Mean | SD |  | Mean | SD |  | p | d |  | p | d |  | p | d |  | p | d |
| **Subchondral Mineralized Plate** | |  |  |  |  |  |  |  |  |  |  |  |  |  |  |  |  |  |  |  |  |  |  |  |  |  |  |
|  | SMP.Th (mm) | 1.694 | 0.411 |  | - | - |  | - | - |  | - | - |  | - | - |  | - | - |  | - | - |  | - | - |  | - | - |
| **Trabecular Bone** | |  |  |  |  |  |  |  |  |  |  |  |  |  |  |  |  |  |  |  |  |  |  |  |  |  |  |
|  | Region.V (mm^3^) | 14,521 | 3,271 |  | 9,726 | 2,420 |  | 6,301 | 1,878 |  | 3,608 | 1,499 |  | 1,535 | 1,216 |  | **< 0.001** | 2.20 |  | **< 0.001** | 1.57 |  | **< 0.001** | 1.23 |  | **< 0.001** | 0.95 |
|  | Tb.vTMD (mg HA/cm^3^) | 412.2 | 21.5 |  | 388.5 | 24.7 |  | 394.7 | 24.9 |  | 411.0 | 26.0 |  | 425.9 | 33.4 |  | **< 0.001** | 0.90 |  | **0.007** | 0.23 |  | **< 0.001** | 0.62 |  | **< 0.001** | 0.56 |
|  | BV/TV (%) | 53.2 | 8.3 |  | 46.6 | 8.3 |  | 52.0 | 9.2 |  | 60.3 | 9.4 |  | 67.0 | 10.6 |  | **< 0.001** | 0.71 |  | **< 0.001** | 0.59 |  | **< 0.001** | 0.90 |  | **< 0.001** | 0.72 |
|  | Tb.N (1/mm) | 0.504 | 0.079 |  | 0.578 | 0.092 |  | 0.610 | 0.085 |  | 0.596 | 0.107 |  | 0.560 | 0.140 |  | **< 0.001** | 0.71 |  | 0.483 | 0.31 |  | > 0.999 | 0.13 |  | 0.135 | 0.36 |
|  | Tb.Th (mm) | 0.807 | 0.141 |  | 0.633 | 0.110 |  | 0.619 | 0.109 |  | 0.659 | 0.106 |  | 0.704 | 0.130 |  | **< 0.001** | 1.45 |  | 0.946 | 0.12 |  | **0.012** | 0.33 |  | 0.445 | 0.37 |
|  | Tb.Sp (mm) | 0.948 | 0.217 |  | 0.943 | 0.190 |  | 0.792 | 0.145 |  | 0.679 | 0.177 |  | 0.605 | 0.181 |  | > 0.999 | 0.02 |  | **0.002** | 0.82 |  | 0.089 | 0.62 |  | 0.410 | 0.40 |
|  | CLV.V (mm^3^) | 570.6 | 517.9 |  | 356.5 | 322.2 |  | 83.1 | 114.9 |  | 22.6 | 49.2 |  | 8.0 | 22.9 |  | 0.524 | 0.77 |  | **0.008** | 0.98 |  | 0.051 | 0.22 |  | > 0.999 | 0.05 |

Data are presented as mean ± standard deviation. p-values with corresponding effect sizes are provided as Cohen’s d for comparisons between two regions (0.2 ≙ small, 0.5 ≙ medium, 0.8 ≙ large effect size). Numbers in bold indicate statistical significance (p < 0.05).

Abbreviations: BV/TV, Bone volume to tissue volume. CLV, Cyst-like void. HR-pQCT, High-resolution peripheral quantitative computed tomography. SMP.Th, Subchondral mineralized plate thickness. SD, Standard deviation. Tb.vTMD, Trabecular volumetric tissue mineral density. Tb.N, Trabecular number. Tb.Sp, Trabecular separation. Tb.Th, Trabecular thickness. V, Volume.
